# Retrospective Performance Analyses of over Two Million U.S. QuantiFERON Blood Sample Results

**DOI:** 10.1128/spectrum.00096-21

**Published:** 2021-07-28

**Authors:** Caixia Bi, Richard B. Clark, Ronald Master, Hema Kapoor, Martin H. Kroll, Ann E. Salm, William A. Meyer

**Affiliations:** a Quest Diagnostics, Secaucus, New Jersey, USA; Fundacio irsiCaixa

**Keywords:** assay performance, indeterminate rates, *Mycobacterium tuberculosis*, positivity rates, QuantiFERON tests, QFT tests

## Abstract

Both the QuantiFERON-TB Gold Plus (QFT-Plus) and the QuantiFERON-TB Gold In-Tube (QFT-GIT) tests are interferon gamma (IFN-γ) release assays (IGRAs) intended to detect *in vitro* cell-mediated immune responses to Mycobacterium tuberculosis antigens. In this study, we retrospectively analyzed performance data for both the QFT-GIT and QFT-Plus test systems from over 2 million samples. QFT-Plus and QFT-GIT testing was performed as specified in the respective package inserts at 23 Quest Diagnostics sites. Blood specimens were collected from individuals in all 50 states from November 2018 through December 2019. Retrospective analyses compared the proportion of positive, indeterminate, and conversion/reversion results. The overall proportion of QFT-positive results was 7% for both the QFT-Plus and QFT-GIT. The proportion of positive results was highest for QFT-GIT (7.5%) followed by the heparin 1-tube QFT-Plus (7.2%); a lower proportion of positives was observed with the 4-tube (all four QFT tubes were used in blood collection) QFT-Plus (6.0%). The proportions of indeterminate results for the 1-tube (heparin-only tube collection) and 4-tube QFT-Plus methods were less than 1% and 4%, respectively. This study indicates a higher proportion of positive results for M. tuberculosis than data from other studies. Additionally, the proportion of indeterminate QFT results were markedly lower when the sample was transported in one lithium-heparin tube instead of direct inoculation into 4 QFT-Plus tubes at the site of blood collection.

**IMPORTANCE** In this study, we retrospectively analyzed results from both the QFT-GIT and QFT-Plus test systems from over 2 million blood specimens. The variables analyzed were (i) QFT positivity rates among various U.S. populations, (ii) indeterminate rates among various types of blood draws and how often an indeterminate result was resolved within 30 days after the initial draw, and (iii) the association of TB1 and TB2 antigen tubes with IGRA reversion and conversion events from serial QFT testing. This is, to our knowledge, the largest QFT study representing patients from an extensive geographic coverage across the United States and U.S. territories.

## INTRODUCTION

Up to one-third of the global population in the world have been infected with Mycobacterium tuberculosis; however, most have latent tuberculosis (TB) infection (LTBI), meaning they do not show signs or symptoms of overt TB disease (1). Approximately 5 to 15% of LTBI patients will manifest M. tuberculosis clinical disease sometime in their lifetime (2). Detection of LTBI is critical for preventing later emergence of clinical disease. Current recommendations suggest that all populations at increased risk for TB be screened for LTBI using either a Mantoux tuberculin skin test (TST) or an interferon gamma release assay (IGRA) (3, 4).

Four generations of the IGRA QuantiFERON (QFT) test have been approved by the U.S. Food and Drug administration since 2001. The newest generation, QuantiFERON-TB Gold Plus (QFT-Plus), became available in 2018, while the previous version, QuantiFERON-TB Gold In-Tube (QFT-GIT), was retired by the manufacturer at that time. These versions measure the *in vitro* IFN-γ cell-mediated immune (CMI) response to M. tuberculosis peptides in a whole-blood sample using an enzyme-linked immunosorbent assay (ELISA)-based system (5, 6). The QFT-Plus test utilizes two different TB-specific antigen-coated tubes instead of the one-tube design used in the prior-generation QFT-GIT (5, 6). The TB1 antigen tube in the QFT-Plus assay system elicits cell-mediated immunity (CMI) responses from CD4^+^ helper lymphocytes, and the TB2 antigen tube elicits CMI responses from both CD4^+^ T-helper and CD8^+^ lymphocytes (5, 7). The TB2 tube may improve sensitivity for TB immune responses, although additional studies are warranted to confirm this.

In this study, we retrospectively analyzed results from the QFT-Plus test systems from over 2 million blood specimens. The variables analyzed were (i) proportion of QFT-positive results among various U.S. populations; (ii) proportions of indeterminate results among various types of blood draws and how often an indeterminate result was resolved within 30 days after the initial draw; and (iii) the association of TB1 and TB2 antigen tubes with IGRA reversion and conversion events from serial QFT testing. This is, to our knowledge, the largest QFT study representing patients from an extensive geographic coverage across the United States and U.S. territories.

## RESULTS

### QuantiFERON positive results.

More than 2.3 million QFT test results were included in this study (Table 1), with 1.4% being repeated specimens from the same patient. A higher proportion of QFT testing was performed using the QFT-Plus method (2.2 million) compared to the QFT-GIT method (0.1 million), as the latter method was being retired (Table 1).

**TABLE 1:** QuantiFERON-TB (1-tube, 4-tube QFT-Plus, and QFT-GIT) positive results

Parameter^*a*^	QFT-Plus (1 and 4-tube combined)	1-tube QFT-Plus	4-tube QFT-Plus	QFT-GIT^*a*^
No. specimens	2.2 million	1.9 million	0.3 million	0.1 million
Total % of positives (95% CI)	7.0 (6.95, 7.02)	7.2 (7.1, 7.2)	6.0 (5.9, 6.1)	7.5 (7.4, 7.7)
% Samples positive by both TB1 and TB2 tubes (95% CI)	85.6 (85.4, 85.7)	86 (85.8, 86.2)	83 (82.4, 83.4)	NA
% Samples positive by TB1 only (95% CI)	6.0 (5.9, 6.1)	5.7 (5.6, 5.8)	8.2 (7.8, 8.6)	NA
% Samples positive by TB2 only (95% CI)	8.4 (8.3, 8.6)	8.3 (8.2, 8.5)	8.9 (8.6, 9.4)	NA

a CI, confidence interval; NA, not applicable.

The overall proportion of positive results for samples assayed using all QFT test versions was 7.0%. The proportion of positive results was significantly lower for the 1- and 4-tube QFT-Plus tests (average 7.0%; 95% confidence interval [CI] 6.95 to 7.02%) than the QFT-GIT test (7.5%; 95% CI 7.4 to 7.7%). The proportion for the single-draw tube QFT-Plus option (7.2%; 95% CI 7.1 to 7.2%) was significantly higher than that of the 4-draw tube option (6.0%; 95% CI 5.9 to 6.1%) (Fig. S2 in the supplemental material).

**FIG 1:**
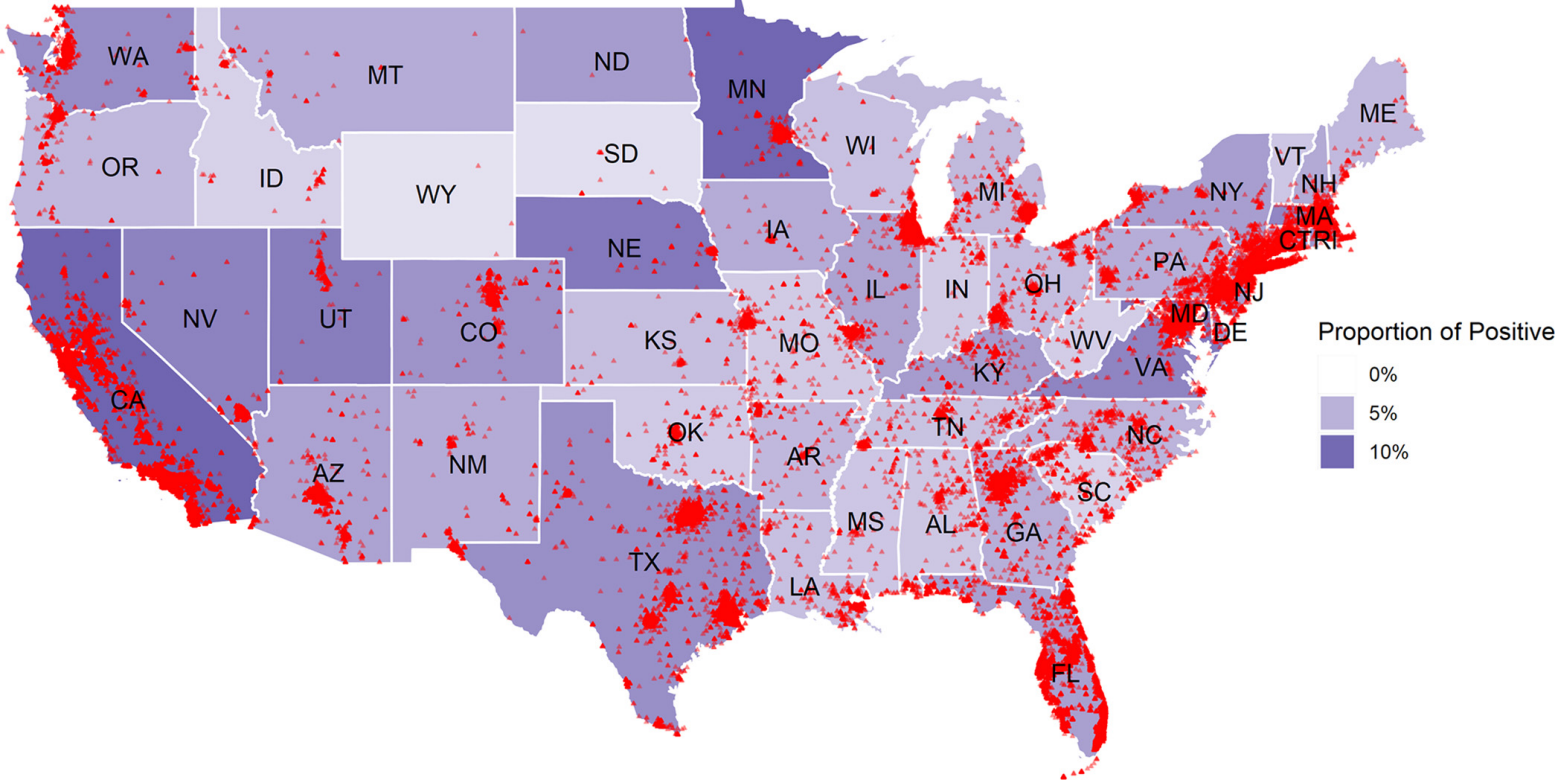
Map of the continental United States showing the proportions of positive QuantiFERON (1-tube, 4-tube QFT-Plus, and QFT-GIT) results based on the number of tests performed. The darker shade intensity indicates a higher proportion for a state. Individual data points represent a QuantiFERON-TB positive patient.

The proportion of positive results varied among states (Fig. 1). Among jurisdictions with more than 300 samples, proportions (based upon number of tests performed, not by population) were highest in California and Minnesota (>10%) and lowest in South Carolina and Idaho (<3%) (Fig. S1). A potential TB “hot spot,” i.e., the proportion of positives for New York City, was 6.0% among 17,482 patients tested; for the District of Columbia, it was 5% among 5,053 patients tested. Fig. S2 and S3 show the proportion of positive results by region, age, gender, and clinical specialty.

Of QFT-Plus-positive samples, 85.6% were positive for both TB1 and TB2 antigens, 6.0% were positive for only QFT-TB1, and 8.4% were positive for only QFT-Plus TB2. The proportion of TB1-only and TB2-only positives was significantly different by the proportion Z test for both 1-tube QFT-Plus and 4-tube QFT Plus options (Table 1).

The proportion of overall (combined QFT-Plus and QFT-GIT) positive results was higher among males (7.6%; 95% CI 7.54 to 7.65%) than females (6.6%, 95% CI 6.56 to 6.65%). This difference held true for both subgroups (1-tube QFT-Plus and 4-tube QFT-Plus).

The proportions of positive results were significantly lower in the 0- to 2-year-old and 3- to 5-year-old age groups (1.4% and 1.2%, respectively) and significantly higher in individuals over 65 (>10%), compared to other age groups (Table 2). These findings held true when the 1-tube QFT-Plus, 4-tube QFT-Plus, and QFT-GIT versions were individually analyzed (data not shown).

**TABLE 2:** QuantiFERON-TB (1-tube, 4-tube QFT-Plus, and QFT-GIT) proportions of positive and indeterminate results by age group

Age group	No. specimens	% Positive (95% CI)^*b*^	% Indeterminate (95% CI)
0–2	11,974	1.4 (1.19, 1.61)	3.4 (3.1, 3.7)
3–5	37,947	1.2 (1.12, 1.35)	1.7 (1.5, 1.8)
6–17	187,928	3.0 (2.88, 3.03)	1.03 (0.98, 1.07)
18–65	1,842,247	7.2 (7.16, 7.23)	1.2 (1.19, 1.23)
>65	230,569	10.0 (9.92, 10.17)	3.0 (2.9, 3.1)
Total^*a*^	2,310,665	7.0 (6.97, 7.04)	1.4 (1.38, 1.41)

a Any records with missing age information are not included.

b CI, confidence interval.

### QFT indeterminate results.

The proportion of indeterminate results for the 1-tube QFT-Plus collection method (0.83%) was 80% lower than that obtained with the 4-tube QFT-Plus method (4.2%) and 69% lower than that obtained with the QFT-GIT method (2.7%).

The predominant cause of indeterminate results was an unacceptably low positive control (mitogen) result (Table 3). The low mitogen values, however, accounted for a lower proportion of indeterminate results for the 1-tube QFT-Plus method (80%) than the 4-tube QFT-Plus (98%) or QFT-GIT (95%; Table 3) tests. The proportion of indeterminate results associated with elevated nil values (>8.0) was significantly higher with the 1-tube QFT-Plus method (total of 20%) than the 4-tube QFT-Plus method (total of 2.4%). Fig. S4 and S5 show the difference between region, age, gender, and the proportion of indeterminate results by clinical specialty.

**TABLE 3:** QuantiFERON-TB indeterminate results

Parameter^*d*^	1-tube-QFT-plus	4-tube-QFT-plus	QFT-GIT
Total no. of samples	1.9 million	0.3 million	0.1 million
Total no. of indeterminate results	15,671	13,938	3,023
Total % indeterminate results (95% CI)	0.83 (0.81, 0.84)	4.2 (4.18, 4.32)	2.7 (2.63, 2.82)
% Indeterminate results due to low mitogen (+ control)^*a*^ (95% CI)	80 (79.5, 80.7)	98 (97.3, 97.8)	95 (93.8, 95.4)
% Indeterminates due to high nil (− control)^*b*^ (95% CI)	12.3 (11.76, 12.79)	1.4 (1.22, 1.62)	3.6 (2.94, 4.27)
% Indeterminates due to high – and high + control^*c*^ (95% CI)	7.6 (7.2, 8.03)	1 (0.84, 1.17)	1.5 (1.03, 1.88)

a Nil tube ≤8 IU/ml and mitogen <0.5 IU/ml.

b Nil tube >8 IU/ml and mitogen <0.5 IU/ml.

c Nil tube >8 IU/ml and mitogen ≥0.5 IU/ml.

d CI, confidence interval.

The frequency of indeterminate results was significantly higher in the pediatric (0 to 2 and 3 to 5 years of age) and elderly populations (>65 years) than in other age strata (Table 2).

### QFT serial testing.

The results of serial QFT-Plus testing are shown in Table 4. Overall, testing of a second specimen resolved the indeterminate result in 64% of cases for specimens collected within 30 days of the initial specimen (Table 4a), and 77% of cases for specimens collected more than 30 days after the initial specimen (Table 4b). Only a small percentage (1%) converted from negative to positive; however, a substantial proportion of patients who were retested after an initial positive result reverted to a negative result on subsequent testing (30% when repeat testing was done within 30 days and 24% when retesting was done more than 30 days after the initial positive result).

**TABLE 4a:** Results of repeat testing within 30 days of the first test on 1-tube and 4-tube QFT-Plus

Result of 1st collection event	No. samples	Result of 2nd collection event (%)		
		Indeterminate	Negative	Positive
Indeterminate	416	36.1	50.0	13.9
Negative	2,134	0.6	98.0	1.4
Positive	1,499	0.5	29.9	69.6

**TABLE 4b:** Results of repeat testing after 30 days of the first test on 1-tube and 4-tube QFT-Plus

Result of 1st collection event	No. samples	Result of 2nd collection event (%)		
		Indeterminate	Negative	Positive
Indeterminate	377	23.3	66.8	9.8
Negative	22,757	0.2	98.5	1.3
Positive	1,688	1.3	24.3	74.4

The proportion of reversion results were substantially higher when only one of the two original QFT-Plus TB antigen test tubes was positive (62.0% for TB1 and 60.3% for TB2) than when both were positive but below 1.0 IU/ml (i.e., 0.35 to 0.99 IU/ml) (Table 5a). The proportion of reversion results was even lower when both TB1 and TB2 antigen tube results from the initial test were ≥1.0 IU/ml (4.4%; 95% CI 2.6 to 6.1%).

**TABLE 5a:** QFT-Plus (1-tube and 4-tube) reversion occurrence in detail for retest within 30 days

Initial QFT values (TB1)	Initial QFT values (TB2)	Total no. of specimens	% Reverting to negative with second blood collection event
<0.35	0.35–0.99	214	60.3
0.35–0.99	<0.35	158	62.0
0.35–0.99	0.35–0.99	469	28.6
0.35–0.99	≥1	63	15.9
≥1	0.35–0.99	41	36.6
≥1	≥1	505	4.4

Similar results were observed when retesting was done more than 30 days after the initial test (Table 5b).

**TABLE 5b:** QFT-Plus (1-tube and 4-tube) reversion occurrence in detail with retest after 30 days

Initial QFT values (TB1)	Initial QFT values (TB2)	Total no. of specimens	% Reverting to negative with second blood collection event
<0.35	0.35–0.99	204	62.3
<0.35	≥1	18	72.2
0.35–0.99	<0.35	115	64.3
0.35–0.99	0.35–0.99	478	31.2
0.35–0.99	≥1	76	14.5
≥1	0.35–0.99	33	9.1
≥1	≥1	804	4.1

## DISCUSSION

This large study showed an overall proportion of positive QFT results of 7% nationwide, with some U.S. states having >10%. This observed overall proportion is higher than other estimates (8). Few specimens had indeterminant results, and most of these could be resolved with a second specimen collection. The proportion of indeterminate results from the QFT-Plus test were significantly lower with the 1-tube than the 4-tube option. For patients with repeat QFT testing, categorical reversions from positive to negative were occasionally observed and may reflect fluctuation near the assay cutoff or other unidentified causes.

### Geographic distribution.

Recent estimates by the Centers for Disease Control and Prevention (CDC) indicate that up to 15 million people in the United States may be infected with TB (9, 10). Our study evaluated greater than 2 million QFT test results and found 7% to be positive even though we were not testing a random population. The CDC estimates of LTBI prevalence in the United States were based on approximately 40,000 TB genotyping cases (3.1 to 5%) (9). In another study, based on the 2011 to 2012 National Health and Nutrition Examination Survey (NHANES), approximately 7,000 individuals were tested by IGRAs and TST (11). The proportions of positive results were lower than those observed in the current study by both IGRA (5% positive) and TST (4.7% positive) testing (11).

In the United States, most clinical TB appears in four states: California, New York, Florida, and Texas (8, 12). The 2018 CDC U.S. TB incidence distribution map appears similar to our distribution map (Fig. 1) in that both California and Minnesota had among the highest TB incidences in the country (12). One reason for the higher proportion of positive QFT results in both California and Minnesota may be a higher proportion of foreign-born individuals in these states (13). Many of the QFT-positive patients could have been exposed to TB in their non-U.S. country of birth.

### Distribution by gender.

In our study, the proportion of positive QFT results was lower in females (6.6%) than males (7.65%) (*P* < 0.001). This observation agrees with the findings of a 2019 U.S. incidence study, which found that males accounted for 60% of incident clinical TB cases (8). Similarly, another study showed that females (4.2%) were statistically less likely than males (5.8%) to be positive on IGRA testing (14). A large systematic review and meta-analysis found that, in other parts of the world, lower- and middle-class men also have a greater prevalence of TB (15). The authors concluded that men may be disadvantaged in seeking and/or accessing TB care in many settings (15). Also, men are likely to remain infectious in the community for longer periods than women (15).

### Assay performance characteristics.

For the QFT-Plus test version, the proportion of TB1-only positive results (8.6%) was significantly higher than that of TB2-only positive results (6%). The TB1 tube in the QFT-Plus test version contains long peptides derived from the mycobacterial antigens designated ESAT 6 and CFP-10; these antigens are thought to primarily elicit a CD4 T-cell response (5). The TB2 antigen tube contains the same components as the TB1 tube and, in addition, peptides that induce CD8 release of IFN-γ (5, 7). These M. tuberculosis-specific CD8^+^ cells have been detected in both active TB and LTBI patients (5, 7). These same M. tuberculosis-specific peptides have also been associated with recently exposed individuals, in an immunocompromised patient population, and in young children (16–18). Serial assessments using both TB1 and TB2 may be helpful in assessing TB conversions (19).

The inclusion of the TB2 version in the QFT-Plus test version could theoretically be responsible for increased assay sensitivity, as this tube is not included in the QFT-GIT test version. Other studies have documented that TB2 antigen tubes alone may be positive more often than TB1 tubes alone (19–21). One review of the literature concluded that QFT-Plus is more sensitive than QFT-GIT for detecting TB infections, mainly because of TB2 responses (22).

Other clinical studies, however, showed no significant difference in the proportion of positive results between TB1 and TB2 tubes (23). Fifteen clinical studies comparing QFT-Plus to QFT-GIT were recently reviewed (24). Comparisons were made between various high- and low-risk TB populations. The authors concluded that, in most studies, QFT-Plus is not able to detect either latent or active disease significantly better than QFT-GIT; thus, the addition of TB2 antigen may not be helpful at this time (22–24). Perhaps larger studies could have shown a statistically significant difference with QFT-Plus versus QFT-GIT. However, as suggested by others, it is possible that some specimens that are positive for only TB1 or TB2 are falsely positive, and that repeat testing may yield a negative result (Table 5) (24). Given the conflicting literature on this topic, further investigation into the role of TB2 in detecting TB may be warranted. Our large study showed that QFT-GIT had a higher proportion of positive results than the later generation, QFT-Plus. This finding could be due to differences in samples of patients within the United States.

Patients with a QFT-indeterminate test result normally need to have the test repeated on a second sample either by a second QFT test or with an alternative IGRA method, such as T-SPOT.*TB* (25). Repeat testing delays proper diagnosis, may be inconvenient for the patient, and increases health care costs. Our data demonstrate that repeat testing is useful in resolving indeterminate results. We found that retesting within 30 days resolves 65% of the initially indeterminate results by providing a subsequent dichotomous positive or negative result value. Repeatedly indeterminate test results from the same patient could in some instances reflect anergy (26), especially if the indeterminate result is due to a lack of response to the positive phytohemagglutinin control.

### Stability and handling.

We observed fewer indeterminate results with the 1-tube QFT-Plus than with the 4-tube QFT-Plus test. Possible reasons for this difference include (i) relatively longer stability of samples collected in the 1-tube QFT-Plus version (48 h) (5) versus the 4-tube QFT-Plus version (16 h) (5); and (ii) 1-tube QFT-Plus tubes are aliquoted in the lab utilizing automated instrument handling and fewer manual preanalytic steps. These data are consistent even when analyzed for age, region, and gender (Fig. S4).

The 4-tube QFT-Plus samples are directly drawn offsite by a remote non-Quest Diagnostics laboratory with stringent collection specifications, including appropriate shaking and processing of the tubes. In this blood collection option, after local blood incubation is completed at 37°C, the site forwards the patient’s plasma to the Quest facility to complete the testing. It is well documented that strict adherence to the QFT package insert instructions during the blood collection event decreases the proportion of indeterminate results (5, 27–29). Shortening the time between blood collection and incubation may also lower the proportion (30). Herrera et al. reported a lower proportion of indeterminate results (<1%) after implementing immediate incubation after blood collection (31).

### Children.

Our data and others indicate that indeterminate results are more common among children than older populations (excluding adults >65 years of age in our study) (32). Impaired immunity has been independently associated with a higher probability of an indeterminate QFT-GIT (33, 34), along with patients with active TB (32). One study suggested that T-cell based assays, such as QFT, have poor performance in children with immature or impaired immune systems (35). A higher proportion of indeterminate results was reported among children less than 2 years of age than among older children (36), while a study by Tebruegge et al. reported that the proportion of indeterminate results was significantly higher in pediatric and elderly (9.1% and 7.4%, respectively) than in adult (2.6%; chi-square test, *P* < 0.0001) patients (37). Others have published low proportions of indeterminate QFT-Plus results among pediatric patients (approximately 2.5%) (23). A study by Zrinski et al. tested >2,000 nonimmunocompromised pediatric patients and demonstrated less than 0.5% indeterminate results (38). Differences in the tested patient populations and the technical competence of the QFT laboratories may account for these apparent contradictions.

### Indeterminate specimen results.

We further assessed gamma interferon levels of nil and mitogen parallel control tube values in 1-tube QFT-Plus and 4-tube QFT-Plus indeterminate samples. Though the proportion of indeterminate results was significantly lower in 1- versus 4-tube QFT-Plus, the proportion of indeterminate results due to high nil values was significantly higher in the 1-tube QFT-Plus method (12%) compared to the 4-tube QFT-Plus method (1.4%). The QFT-Plus package insert states that, in clinical studies, less than 0.25% of subjects had background IFN-γ levels of >8.0 IU/ml (5). The higher background levels could indicate the presence of heterophile antibodies, or intrinsic IFN-γ secretion (5). In a large study by Banach et al. (*n* = 28,864; 2,058 [7%] positive; 522 [2%] indeterminate), 264 (50.5%) of the indeterminate results were due to low mitogen, while 258 (49.4%) of the indeterminate results were due to a high nil tube value (39).

### Occurrence of reversion and conversion.

The reversion of a positive QFT test to a negative test (or vice-versa, a conversion from negative to positive) on serially collected samples from the same person is called the “wobble” effect by some investigators (40). This wobble effect is frequently observed, as shown here (30% of the positive samples retested within 30 days of the first draw were negative on the second specimen collection event) and in other QFT studies (26, 41), and may be due to an unstable immune response and/or an assay-related variability. Lewinsohn et al. recommend that all positive IGRA tests be repeated from patients who have a low pretest probability of a TB infection (26). Thanassi et al. stated that clinicians should retest low-risk individuals with initial QFT results of <1.11 IU/ml (42). Also, some researchers have recommended an indeterminate (equivocal) or uncertain zone be created around the assay cutoff point (41, 43). These zones have been proposed to range from 0.2 to 0.7 IU/ml (35) or 0.2 to 0.99 IU/ml (36); use of these zones or ranges could allow for a more reliable and valid assay that may better definitely detect patients with TB infections. Our data indicate that >95% samples that showed a repeat positive result on serially collected sample when both the TB1 and TB2 antigen tubes had a value of ≥1.0 IU/ml.

One strength of this study lies in the sample size and heterogeneity of the geographic distribution of the collected samples. The overall U.S. observed proportion of positive results is approximately 7% in over 2 million QFT tests.

A limitation to our data could be a high positive-sample bias due to accessibility of national reference lab patient services centers (PSCs) located, predominantly, in large urban centers, where greater numbers of high-risk populations (homeless, immunocompromised, foreign borne, or HIV-positive patients, etc.) are likely to reside. Likewise, sample bias may be introduced by underrepresentation of states with low proportions of positive QFT results (e.g., South Dakota and Wyoming) in our data set, compared to national TB/LTBI estimates (8, 12). In addition, the test results obtained in this study could not be correlated with patient history or other patient diagnostic test modalities (e.g., chest radiographic imaging). Higher-risk groups, such as foreign-borne patients, may have been more likely to be tested. The temporal bias of testing (the QFT-Git was being phased out) could also affect the data. Analysis could also be affected by other potentially confounding factors that were neither identified nor controlled for in this retrospective evaluation of test results. In addition, the proportion of positive QFT and indeterminate results appears to be dependent upon the clinical specialty of the site where they were collected (Fig. S3 and S5).

In conclusion, this large national QFT study suggests that TB positivity rates in the United States may be higher than previous estimates. In addition, indeterminate QFT-Plus results are markedly less common when the sample is transported by a single lithium-heparin tube rather than direct inoculation into 4-QFT-Plus tubes. Most indeterminate QFT-Plus results can be resolved by a repeat sample collection within a month or later. Finally, when both the TB1 and TB2 antigen tubes of a QFT-plus test are ≥1.0 IU/ml, a positive result is much more likely (>95%) to be confirmed as such on repeat specimen collection testing.

## MATERIALS AND METHODS

Results from consecutive patient QFT tests performed from November 2018 through December 2019 were extracted from the Quest Diagnostics Informatics database. Quest Diagnostics is a national clinical reference laboratory in the United States; patient results were collected from all 50 states, Washington, DC, Puerto Rico, Guam, the Virgin Islands, Northern Mariana Islands, and several U.S. military jurisdictions.

All QFT test procedures and categorical interpretation of the test results (positive, indeterminate, or negative) were performed and interpreted as outlined in the QFT package inserts (5, 6). All results were categorized and tabulated according to one of these 3 categories (Table 2). Most of the QFT-GIT data were collected from Nov 2018 to Mar 2019, as the vendor stopped supplying reagents for this assay owing to the presence of the new assay, QFT-Plus. The current package insert (QFT-Plus) states that the QuantiFERON test has not been extensively evaluated in individuals younger than age 17 years.

For the QFT-Plus assay, there are two options for each individual blood collection event; one option used a single lithium-heparin anticoagulated blood tube. With this option, aliquots of the lithium-heparin whole-blood sample are subsequently transferred at the testing laboratory within 48 h to four QFT-Plus Blood Collection Tubes (1-tube QFT-Plus). The second QFT-Plus blood sample collection option is to directly collect the specimen into four QFT-Plus dedicated collection blood tubes (4-tube QFT-Plus). With the prior QFT-GIT test version, the blood specimen is directly collected into three QFT-GIT tubes.

Retrospective data analyses of results for the above three QFT blood collection methods were conducted. Statistical significance of comparisons was evaluated using 95% confidence intervals or the proportion Z-test. The study was deemed exempt by the Western Institutional Review Board (Puyallup, WA); all data were only analyzed in aggregate categories.

### Data availability.

The data are stored in the Quest Diagnostics Data Informatics Warehouse and are extractable upon request in compliance with HIPAA. HIPAA clearly defines research use of data as analyzed for this and numerous other studies based on 45 CFR 164.501, 164.508, 164.512(i) (see also 45 CFR 164.514[e], 164.528, 164.532). Analyses were performed using R, version 3.6.1 (R Project for Statistical Computing).
