# Supplementary material for: Retrospective Performance Analyses of over Two Million U.S. QuantiFERON Blood Sample Results
Source: Microbiol Spectr. 2021 Jul 28;9(1):10.1128/spectrum.00096-21. doi: 10.1128/spectrum.00096-21 (PMC8552680; doi:10.1128/spectrum.00096-21)

Online Supplementary Material

Supplemental Fig. 1. QFT proportion of positive results by state

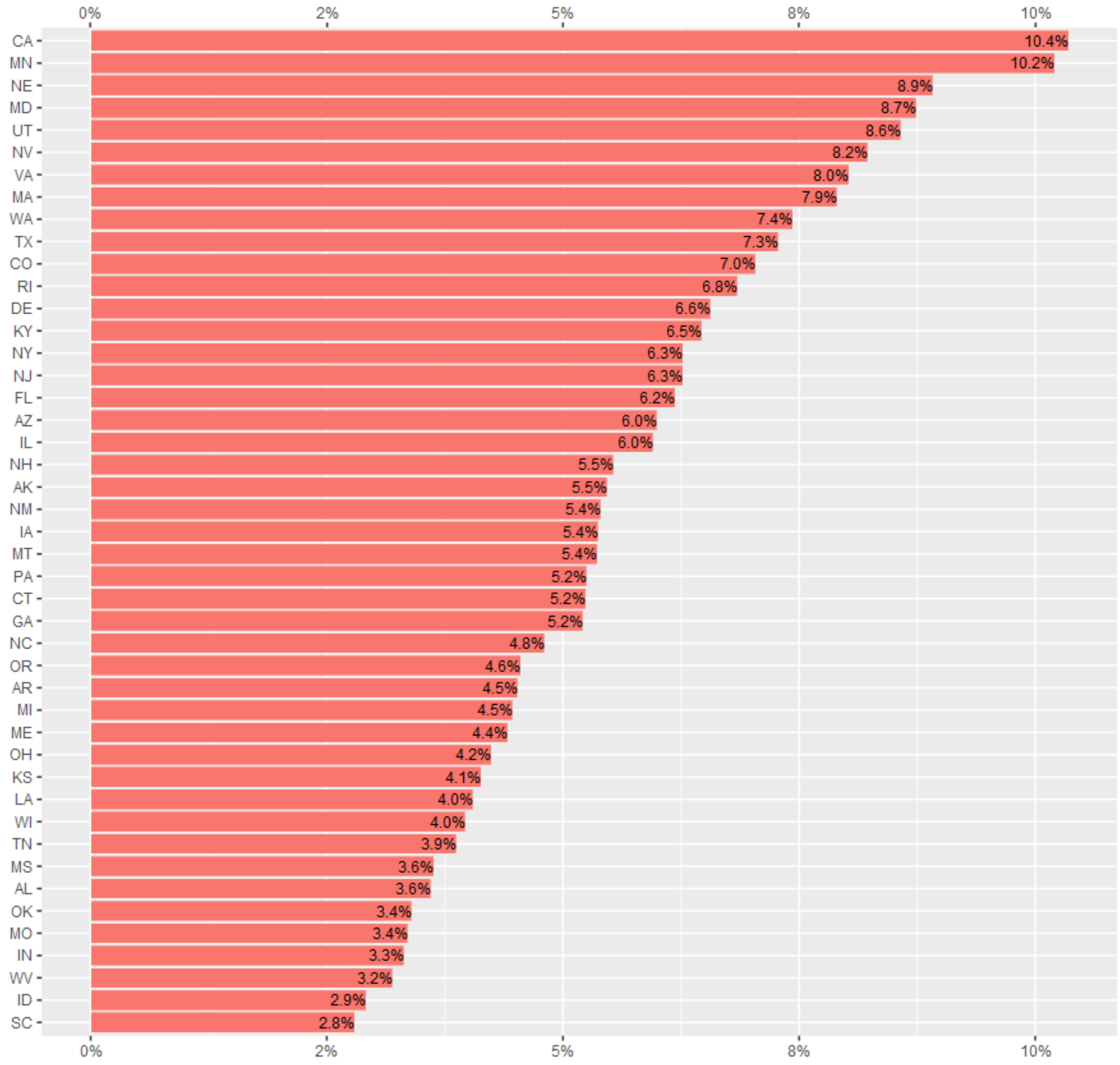

Supplemental Fig. 2. Proportion of positive QFT results by method, region, age, and gender. 1T represents QFT-Plus with one heparin tube draw, whereas 4T represents QFT-Plus with a four-tube draw; 3T represents QFT-GIT

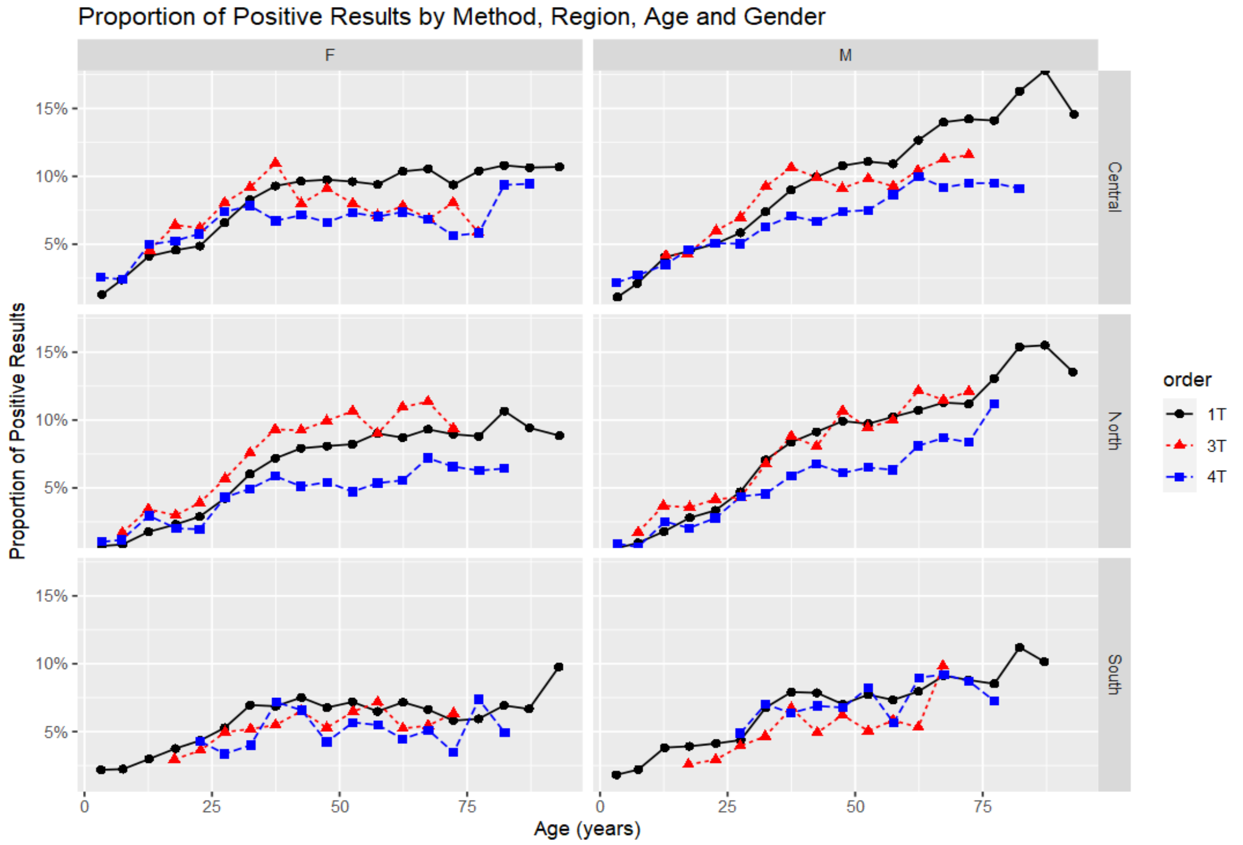

Supplemental Fig. 3. Proportion of positive QFT results by clinical specialty. Most listed specialties had ≥1,000 tests; a minimum number of tests for this figure was 100 tests per specialty. 1T represents QFT-PLUS with one heparin tube draw, whereas 4T represents QFT-Pus with a four-tube draw; 3T represents QFT-GIT.

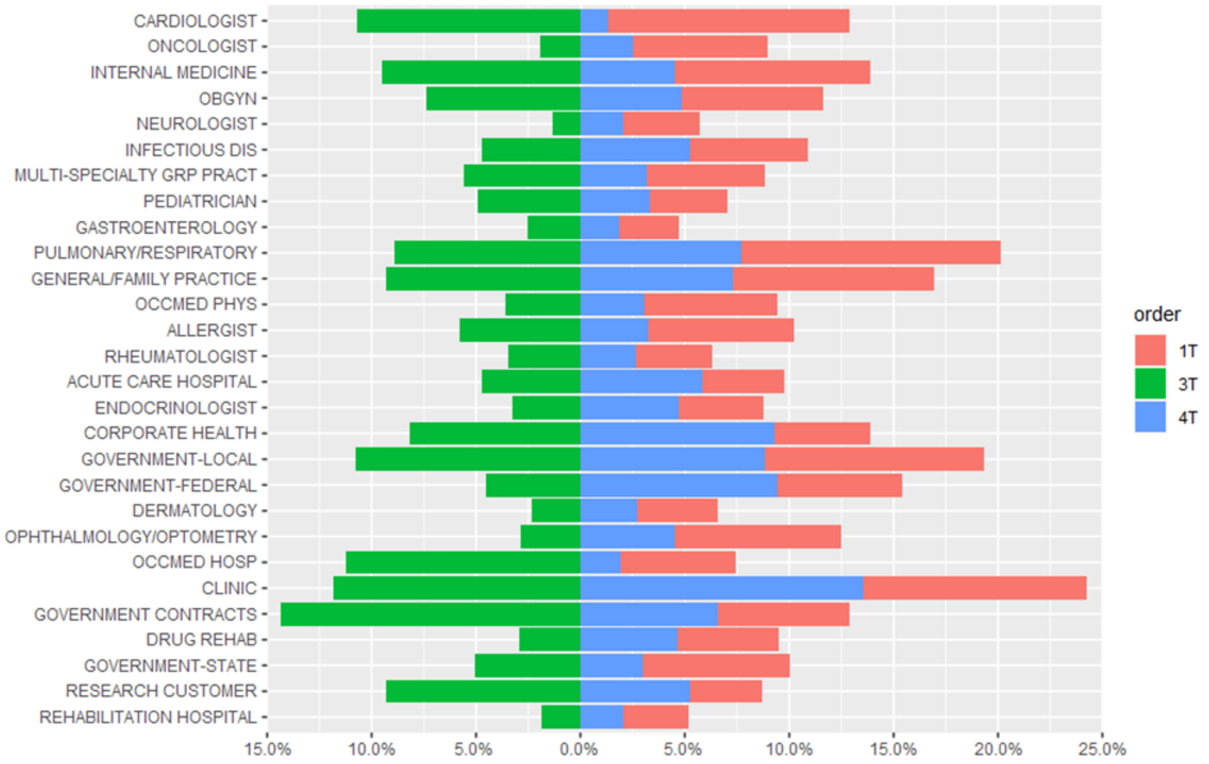

Supplemental Fig. 4. Proportion of indeterminate QFT results by method, region, age, and gender. 1T represents QFT-PLUS with one heparin tube draw, whereas 4T represents QFT-Plus with a four-tube draw; 3T represents QFT-GIT

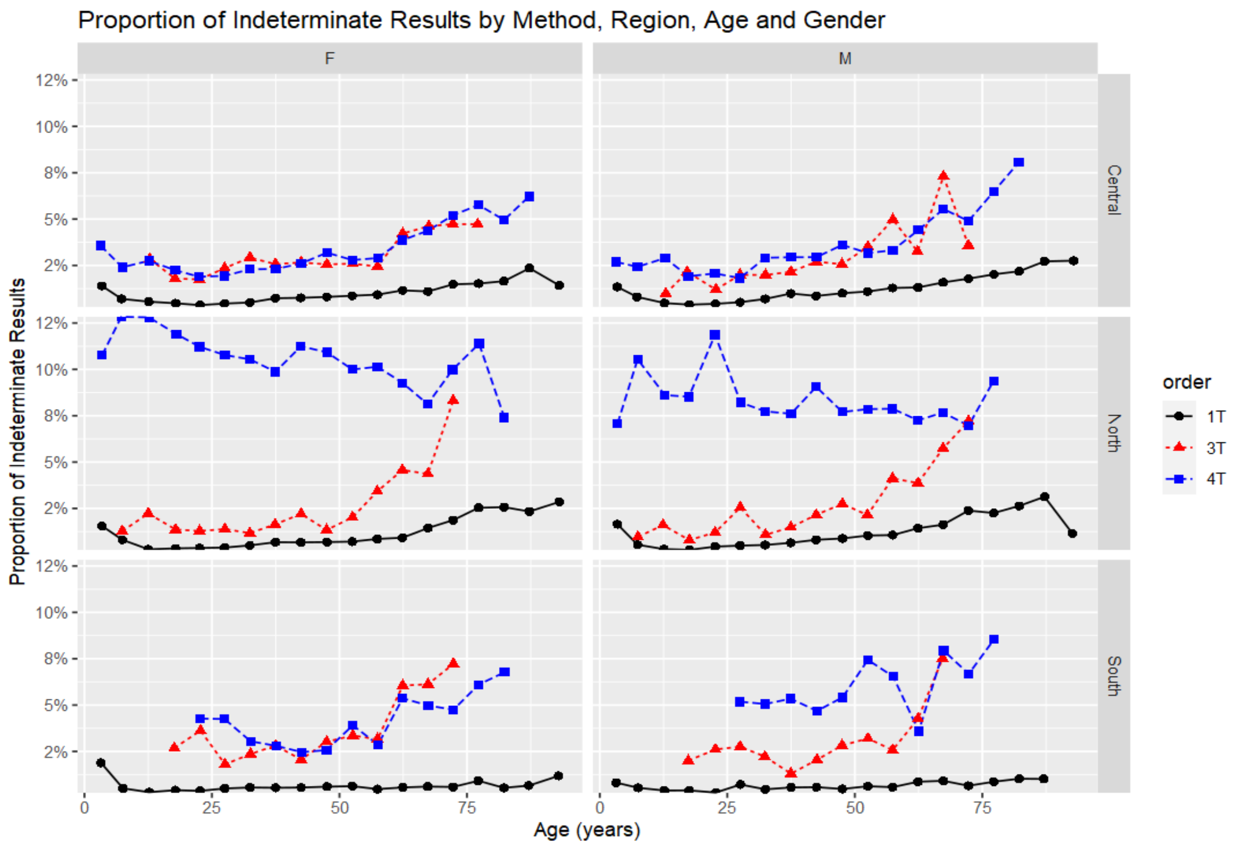

Supplemental Fig. 5. Proportion of indeterminate QFT results by clinical specialty. Most listed specialties had >1,000 tests; a minimum number of tests for this figure was 100 tests per specialty. 1T represents QFT-PLUS with one heparin tube draw, whereas 4T represents QFT-Pus with a four-tube draw; 3T represents QFT-GIT.

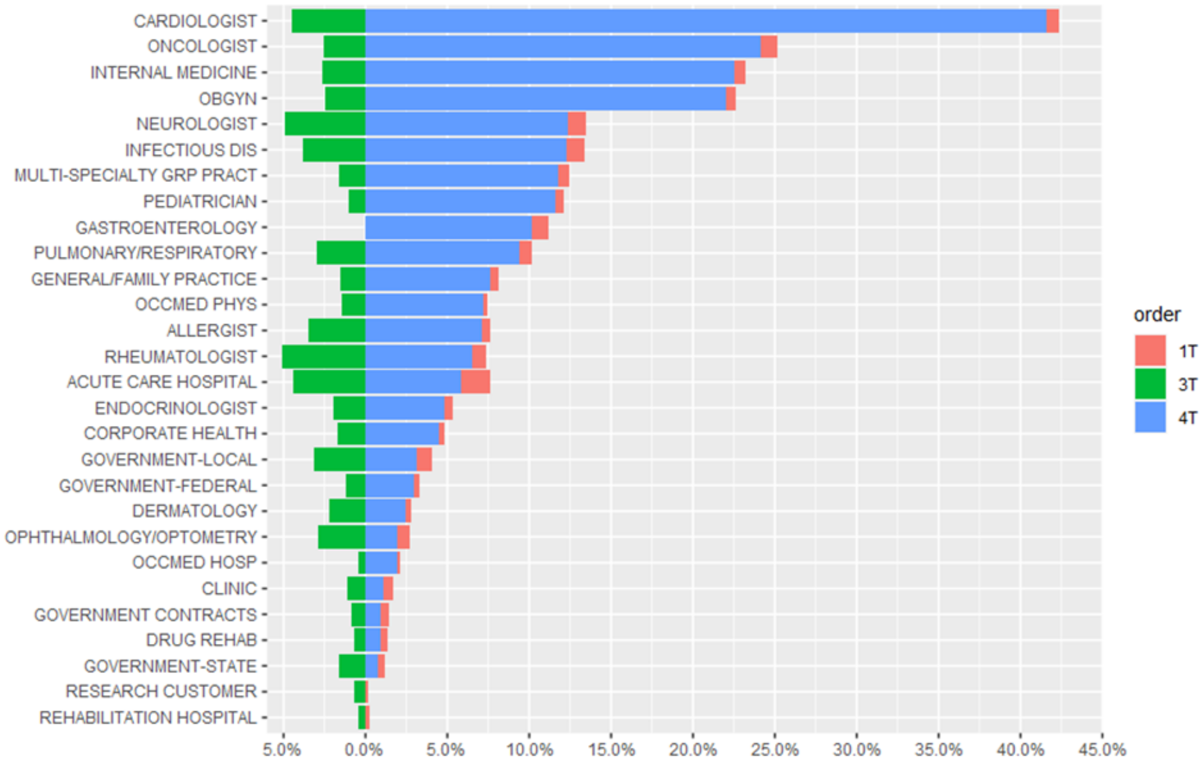

Supplement: SUPPLEMENTAL FILE 1 — Supplemental material. Download SPECTRUM00096-21_Supp_1_seq2.pdf, PDF file, 0.6 MB [file spectrum00096-21_supp_1_seq2.pdf]
